# FT-IR Microspectroscopy of Rat Ear Cartilage

**DOI:** 10.1371/journal.pone.0151989

**Published:** 2016-03-25

**Authors:** Benedicto de Campos Vidal, Maria Luiza S. Mello

**Affiliations:** Department of Structural and Functional Biology, Institute of Biology, University of Campinas (Unicamp), 13083–862, Campinas, SP, Brazil; University of Quebec at Trois-Rivieres, CANADA

## Abstract

Rat ear cartilage was studied using Fourier transform-infrared (FT-IR) microspectroscopy to expand the current knowledge which has been established for relatively more complex cartilage types. Comparison of the FT-IR spectra of the ear cartilage extracellular matrix (ECM) with published data on articular cartilage, collagen II and 4-chondroitin-sulfate standards, as well as of collagen type I-containing dermal collagen bundles (CBs) with collagen type II, was performed. Ear cartilage ECM glycosaminoglycans (GAGs) were revealed histochemically and as a reduction in ECM FT-IR spectral band heights (1140–820 cm^-1^) after testicular hyaluronidase digestion. Although ear cartilage is less complex than articular cartilage, it contains ECM components with a macromolecular orientation as revealed using polarization microscopy. Collagen type II and GAGs, which play a structural role in the stereo-arrangement of the ear cartilage, contribute to its FT-IR spectrum. Similar to articular cartilage, ear cartilage showed that proteoglycans add a contribution to the collagen amide I spectral region, a finding that does not recommend this region for collagen type II quantification purposes. In contrast to articular cartilage, the symmetric stretching vibration of –SO_3_^-^ groups at 1064 cm^-1^ appeared under-represented in the FT-IR spectral profile of ear cartilage. Because the band corresponding to the asymmetric stretching vibration of –SO_3_^-^ groups (1236–1225 cm^-1^) overlapped with that of amide III bands, it is not recommended for evaluation of the –SO_3_^-^ contribution to the FT-IR spectrum of the ear cartilage ECM. Instead, a peak (or shoulder) at 1027–1016 cm^-1^ could be better considered for this intent. Amide I/amide II ratios as calculated here and data from the literature suggest that protein complexes of the ear cartilage ECM are arranged with a lower helical conformation compared to pure collagen II. The present results could motivate further studies on this tissue under pathological or experimental states involving ear cartilage.

## Introduction

Fourier transform-infrared (FT-IR) studies on cartilaginous tissues have aimed to generate information regarding articular cartilage as motivated mostly by clinical implications [1–7]. Due to alterations in the composition and histology of articular cartilage caused by damage and degeneration progressing to osteoarthritis, it is understandable that FT-IR research concerning this specific tissue has garnered increasing interest. Researchers hope to obtain information on healthy tissues to understand the aspects introduced by pathological processes at the molecular level.

The contribution of carbohydrates and, particularly, the application of the second-derivative of the FT-IR spectra have been used to obtain consistent results for articular cartilage studies [2, 5, 6]. Three spectral characteristics have been considered important for obtaining information on this subject: 1) the integrated absorbance in the spectral window profile assigned to the vibrational frequency range of carbohydrates; 2) the carbohydrate/amide I ratio; and 3) the second-derivative peak determined at 1062 cm^-1^ [6]. The second-derivative spectroscopy was considered suitable for the investigation of proteoglycans (PrG) and for improving overall analysis. Also based on FT-IR studies, the amount of collagen and PrG has been reported to vary with cartilage site, age, osteoarthritis progression and species (human versus bovine tissues) [6].

For PrG analysis of articular cartilage, the 1140–985 cm^-1^ spectral region, assigned to contribution of carbohydrate radicals, and the peaks at 1376 cm^-1^, assigned to all acidic glycosaminoglycans (GAGs) and glycoproteins (-CH_3_ symmetric bending vibration), and at 1064 cm^-1^, related to sulfated GAGs (-SO_3_^-^ symmetric stretching vibration), have been found to be particularly relevant [5, 7]. The amide I band absorbance, traditionally representative of collagen content in the articular cartilage, was also shown to be significantly influenced by PrG [5].

Despite these afore-mentioned studies, FT-IR analyses of cartilage models different from articular cartilage in terms of morpho-physiological, biomechanical and biochemical properties are, to the best of our knowledge, still lacking. In particular, this lack of information relates to ear cartilage, which is structured as a thin layer of only a few cells in thickness [8, 9]. This cartilage is also implicated in phenomena that demand physiological/pathological interests, such as the growth factor influence on cartilage wound repair, recovery from thermal injury, destruction of cells by auto-aggressive action and auricular chondritis [10–12]. In addition, because collagen type II contributes significantly to the composition of the articular cartilage extracellular matrix (ECM) [2], it would be interesting to evaluate whether this contribution extends to the FT-IR spectrum of the ear cartilage ECM. Other important questions include what changes are introduced in the FT-IR spectral profile of the ear cartilage ECM after hyaluronidase treatment and whether the spectral profile of the ear dermis, which contains collagen type I, differs from that of pure collagen type II.

The purpose of the present study was to examine the FT-IR properties of rat ear cartilage under healthy conditions. Optical anisotropy of rat ear cartilage ECM and collagen bundles (CB) and of a collagen type II standard was also studied to determine the level of supramolecular organization of their components.

## Materials and Methods

### Materials

The Multidisciplinary Center of Biological Investigation of the University of Campinas (Cemib/Unicamp, Campinas, Brazil) provided Wistar male adult rats. The rat ears used in this investigation were obtained from five specimens previously employed in studies on the supraorganization of collagen bundles in the skin [13] and FT-IR microspectroscopy of elastin and collagen fibers [14]. The protocol for animal care and use (no. 2700–1) was approved and performed in compliance with the Committee for Ethics in Animal Use of the University of Campinas (CEUA/IB/Unicamp, Brazil) and in accordance with the Guidelines of the Canadian Council on Animal Care. The rats were reared under standard controlled conditions, fed extruded chow (Nuvital, Colombo, Brazil) and received water ad libitum.

Both ears from each specimen were removed close to their insertion points and then sectioned from the top to the base. The materials were fixed in 4% paraformaldehyde in 0.1 M phosphate buffer at pH 7.4 for 3 h under vacuum and then stored for 24 h in the fixative solution in the refrigerator. Next, they were rinsed for 24 h in distilled water and processed for embedding in Histosec^®^ without DMSO (Merck, Darmstadt, Germany) at 56–58°C. It was assumed that the paraffin embedding had no effect on the anisotropic and microspectroscopic properties of the collagen bundles [13, 15]. Sections were cut at 7-μm thickness using a Microm HM 315 microtome (Waldorf, Germany). After being dewaxed and hydrated, the sections to be used for optical anisotropy and FT-IR studies remained unstained. Sections to be used for GAG histochemistry were subjected to toluidine blue staining.

Collagen type II was isolated after extraction from chicken sternum cartilages obtained from a local abattoir. Ideally, rat cartilage collagen type II might be used. However, several operational difficulties in isolating or purchasing this collagen type from rats made such an option inadequate for the present purposes.

The chondroitin-4-sulfate sodium salt standard (4-CIS) was obtained commercially (Sigma-Aldrich, St. Louis, USA) and prepared for FT-IR studies as dried smears on glass slides.

### Collagen type II preparation

Briefly, isolated and cleaned sternum cartilage was cut into small fragments on a glass surface over ice to keep the temperature at approximately 0°C. Next, the cartilage fragments were treated with pure acetone, dried at 37°C, and then subjected to papain digestion (40 mg/g tissue) in a solution containing 0.03 M sodium citrate buffer at pH 3.5, 0.04 M EDTA and 0.08 M beta-mercaptoethanol [16, 17]. After PrG removal, the product was rinsed in 1 to 2 L of distilled water and then treated with a 3% acetic acid solution containing 1 mg/g pepsin and 0.5 mL HCl, under agitation, for at least three days. This solution was filtered through a 335-μm sieve and centrifuged. The resulting supernatant was subjected to treatment with a 10% solution of NaCl for collagen II precipitation. The precipitate was separated and subjected to dialysis until a highly viscous, almost transparent collagen type II birefringent mass was obtained. This mass was lyophilized (Lyophilizer Liobras, São Carlos, SP, Brazil) and kept in the refrigerator until further use.

### GAG histochemistry

Toluidine blue staining was used for analysis of the morphological and histochemical aspects of PrG GAG elements in ear preparations. Ear sections were stained with a 0.025% dye (Carlo Erba, Milan, Italy) solution in MacIlvaine buffer at pH 4.0 for 30 min, followed by rinsing in MacIlvaine buffer at pH 4.0 for 15 min and distilled water. The sections were then treated with a 4% ammonium molybdate (Merck, Rio de Janeiro, Brazil) aqueous solution for 4 min and rinsed in distilled water [15, 18]. Next, the samples were air dried, cleared in xylene and mounted in Eukitt (O. Kindler GmbH & Co., Freiburg, Germany). Prior to toluidine blue staining some sections were treated with 1 mg/mL testicular bovine hyaluronidase (Sigma-Aldrich Chemie, Steinheim, Germany) in 0.9% saline [5, 19] for 18 h at 37°C for GAG digestion.

### Optical anisotropy

Analysis of non-compensated birefringence was performed for unstained and toluidine blue-stained ear sections, using an Olympus BX51 polarized light microscope (Olympus America, Center Valley, PA, USA) equipped with a Q-color 5 camera (Olympus America). Unstained sections immersed in distilled water were used to detect total birefringence, i.e., intrinsic and form birefringence, in ECM and CB. Images of the birefringence intensity brilliance were captured and analyzed with Image-Pro Plus 6.3 (Media Cybernetics, Inc. Silver Spring, MD, USA), as previously described [20, 21]. Birefringence sign was confirmed using the 1/4 λ Sénarmont’s compensator [20, 21].

### FT-IR microspectroscopy

FT-IR spectral profiles were obtained for the ear cartilage ECM, pure collagen type II, ear dermal CBs and 4-CIS, using an Illuminat IR II^TM^ microspectroscope (Smiths Detection, Danbury, CT, USA) equipped with a liquid-nitrogen-cooled mercury-cadmium-telluride detector and the Grams/AI 8.0 spectroscopy software (Thermo-Electron Co., Waltham, USA). An Olympus (Olympus America) microscope with attenuated total reflection diamond objective (ATR, magnification 36 x) was part of the equipment. Performance validation of the equipment was indicated by a low signal-to-noise ratio of 7929:1 [22].

Prior to the FT-IR analysis, the cartilage sections were examined using an Olympus BX51 microscope equipped for polarized light studies, with the object to identify ear regions exhibiting birefringence and to document particular zones to be measured at the microspectroscope.

The measurement site area was a square of 25 μm per side. Absorbances were evaluated in the 4000–650 cm^-1^ wavenumber spectral range, with a spectral resolution of 4 cm^-1^ as informed by the equipment supplier. The absorbances of the materials and background were evaluated using 64 scans for each individual spectral profile. At least 10 spectral profiles were obtained from each material. Baseline and level-plus-zero correction were accomplished for each spectrum using the OFF SET.AB application of the Grams software. Normalization to the highest absorption peak of the spectra (in the present case, at 1632–1631 cm^-1^) was performed for each spectrum following instructions provided by Grams software (Function.AB program). An average spectrum was then determined for each sample. Some spectra were also obtained in which the normalization procedure with respect to the 1632–1631 cm^-1^ peak was not performed to show changes in amide I and II spectral regions introduced by hyaluronidase treatment.

An absorbance ratio (AbRCAm) relating the highest absorbance values in regions of carbohydrate and amide I vibration groups as well as an area ratio (ArRCAm) relating areas under specific spectral bandwidths that correspond to these vibration groups were obtained in hyaluronidase-treated and untreated samples using the Numerical Integral Statistics feature provided by the Grams software (AB Program, Spectral Math).

Savitzky-Golay’s 2nd-derivative spectra were obtained to confirm the frequency positions of specific peaks in the 1100–750 cm^-1^ spectral window for cartilage ECM and collagen type II. Peak fitting using a Gaussian function at a medium sensitivity level, following the software instructions, was applied to the amide I spectral region for comparison between hyaluronidase-treated and untreated cartilage ECM.

## Results

### Morphology, GAG evidence and optical anisotropy

**Fig 1:**
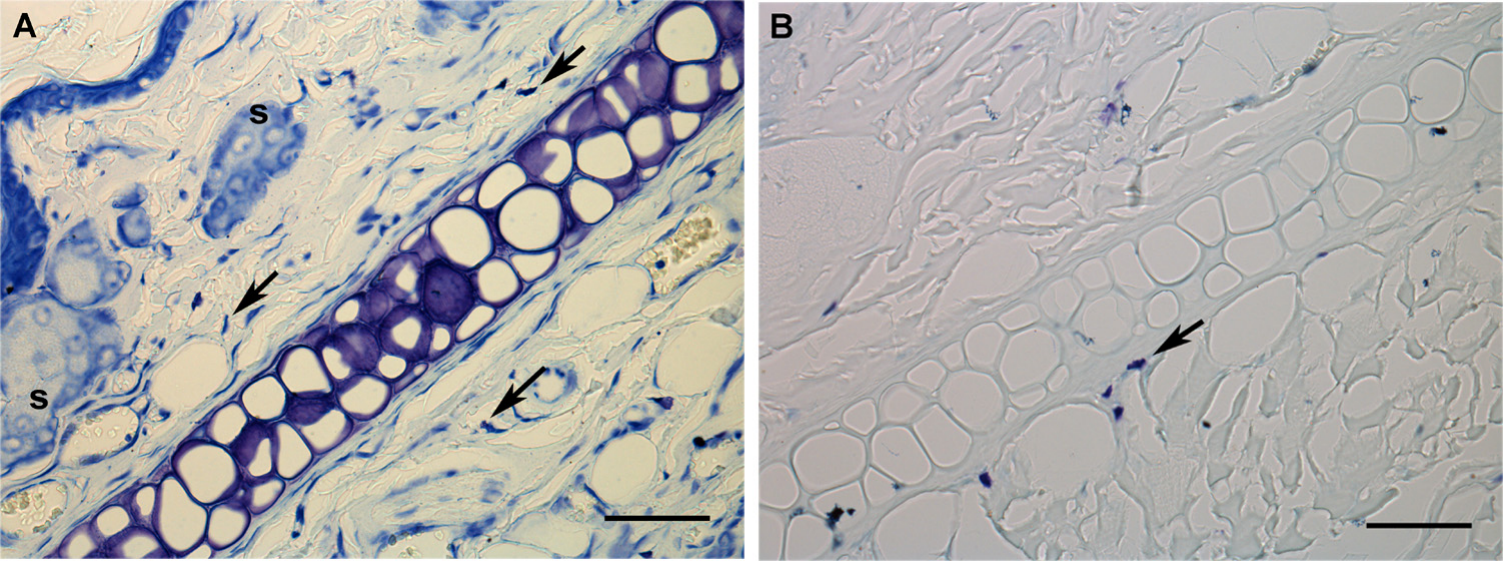
Toluidine blue staining in ear sections subjected (B) or not (A) to hyaluronidase digestion. The arrows indicate mast cells. S, sebaceous glands. Bar = 50 μm.

The ear cartilage was identified as a band halfway between the dermal layers (Fig 1A and 1B). The dermis of the inner face of the ear appeared thinner than that of the outer face of the ear. The cartilage was found to form a layer ~2–3 cells thick extending intradermally. In toluidine blue-stained sections, the ECM surrounding chondrocyte spaces exhibited an intense metachromasy (Fig 1A) that appeared abolished by treatment with hyaluronidase prior to staining, thus revealing GAGs. This treatment did not affect the strong metachromatic reactivity due to heparin in the cytoplasm of mast cells (Fig 1A and 1B).

**Fig 2:**
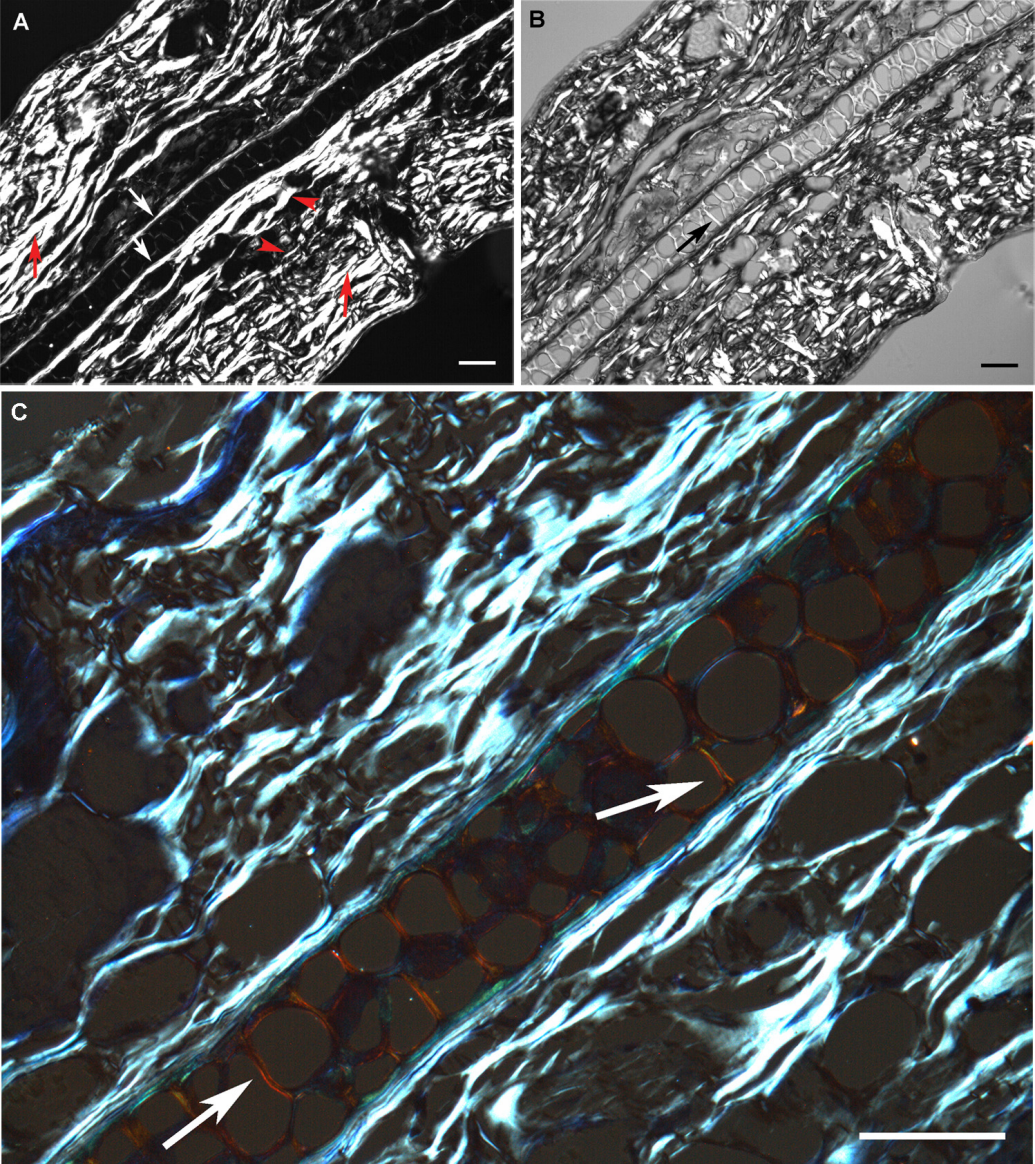
Images of cartilage ECM and CB birefringence in unstained (A, B) and toluidine blue-stained ear sections (C). In A, birefringence intensity is dependent on the orientation and degree of aggregation of CBs (red arrows); white arrows indicate the perichondrium (collagen type I), and arrowheads indicate perichondrium coalescent collagen fibers. In B, the same image as in A, a partial compensation of birefringence is shown in CBs and perichondrium oriented from the SW to the NE direction. Completely compensated birefringence is demonstrated by black color. The most densely aggregated fibers do not exhibit total birefringence compensation. Birefringence of cartilage ECM regions (black arrow) and CBs distributed with their long axis oriented from the SE to the NW direction was not compensated. In C, abnormal interference colors of birefringence are observed in the cartilage. The white arrow indicates cartilage regions with enhanced birefringence intensity due to a Cotton effect-like phenomenon [15, 23]. In this case, no birefringence compensation was performed. Bars = 50 μm.

An intense birefringence in the CBs and, comparatively, a weak birefringence in the cartilage ECM were observed in unstained ear sections immersed in water and examined under polarized light. This could only be observed when the long axes of the CBs, perichondrium and cartilage strip were positioned at 45 degrees with respect to crossed polarizers (Fig 2A and 2B). Variation in the birefringence intensity was due to differences in the direction of the long axis of the aforementioned structures with respect to the crossed polarizers’ directions and the degree of compactness/concentration and composition of the oriented macromolecules involved. Using Sénarmont’s 1/4 λ compensator, it was possible to detect partial birefringence compensation on CBs. As a result, different brilliance intensities were observed in collagen fibers (Fig 2B). The same consideration applies to the perichondrium. The ECM structures positioned perpendicularly to the long axis of collagen fibers and cartilage strip showed enhanced birefringence brilliance intensity (Fig 2B).

In toluidine blue-stained ear sections examined under crossed polarizers, abnormal interference colors of birefringence derived from the abnormal dispersion of the refractive index in the stained substrate were observed only in the cartilage (Fig 2C). Cartilaginous regions distributed with their long axis perpendicular to the perichondrium exhibited enhanced birefringence brilliance intensity (Fig 2C).

The lyophilized collagen type II preparations presented a very soft and light sponge-like structure that, once unpacked, revealed very thin crossed fibrils (S1 Fig) of positive birefringence sign.

### FT-IR spectral characteristics

In this study, particular emphasis was placed on results revealing the contribution of vibration of collagen type II and carbohydrate groups to the IR spectrum and on the 4 cm^-1^ spectral resolution intrinsic to the equipment performance, when evaluation of likely variations in peak wavenumber determination was undertaken.

**Fig 3:**
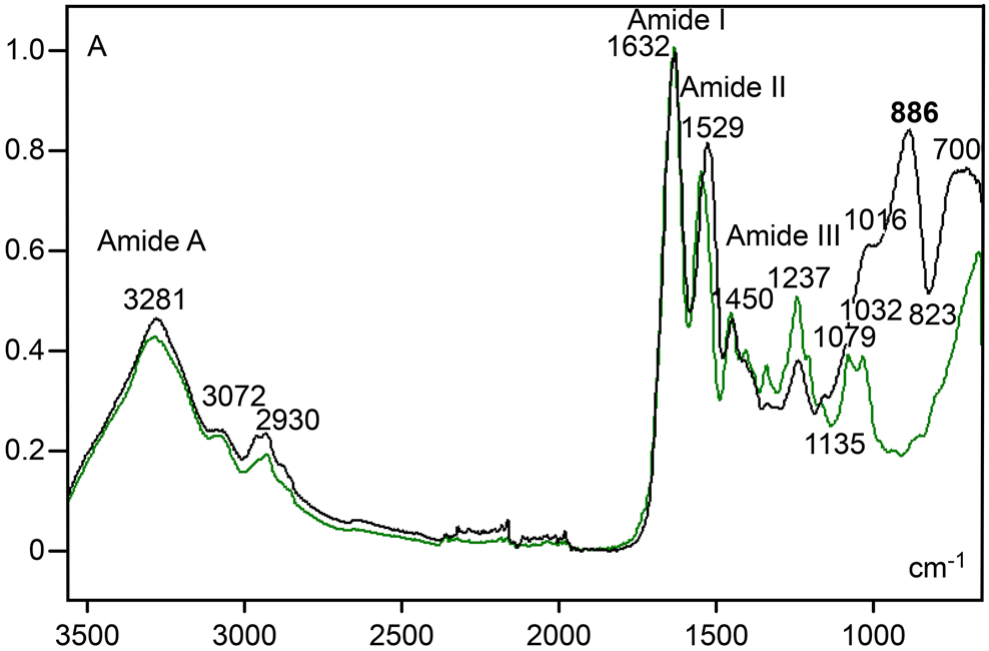
FT-IR spectral profiles for cartilage ECM (black line) and pure collagen type II (green line). See text for details. A, absorbances.

Amide I, II and III band regions were evident in the spectral profile of the cartilage ECM and pure collagen type II samples (Fig 3). The same result was obtained for the amide A band at ~3280 cm^-1^ and band peaks in the ~3070–2920 cm^-1^ spectral region, usually assigned to hydrogen bonding and stretching vibrations of –CH_2_ and –CH_3_ groups, respectively [24]. The amide I/amide II ratio of the cartilage ECM (X = 1.23, S = 0.04), based on calculation from absorbances at 1632 cm^-1^/1529-1528 cm^-1^, was lower than that of pure collagen type II (X = 1.36, S = 0.08), based on calculation from absorbances at 1630 cm^-1^/1547 cm^-1^. Striking differences were found when comparing the contribution of cartilage ECM with that of collagen type II in the carbohydrate wavenumber range of 1140–820 cm^-1^ (Fig 3). The cartilage ECM spectral profile showed a shoulder at 1032–1016 cm^-1^ and an elevated peak at 890–886 cm^-1^, whereas the spectrum for collagen type II showed distinct low peaks at 1079 and ~1031 cm^-1^. Using a ratio corresponding to carbohydrate groups to amide I heights (886 cm^-1^/1632 cm^-1^), the value for the cartilage ECM (X = 0.81, S = 0.08) was found to be higher than that for collagen type II (X = 0.20, S = 0.04).

**Fig 4:**
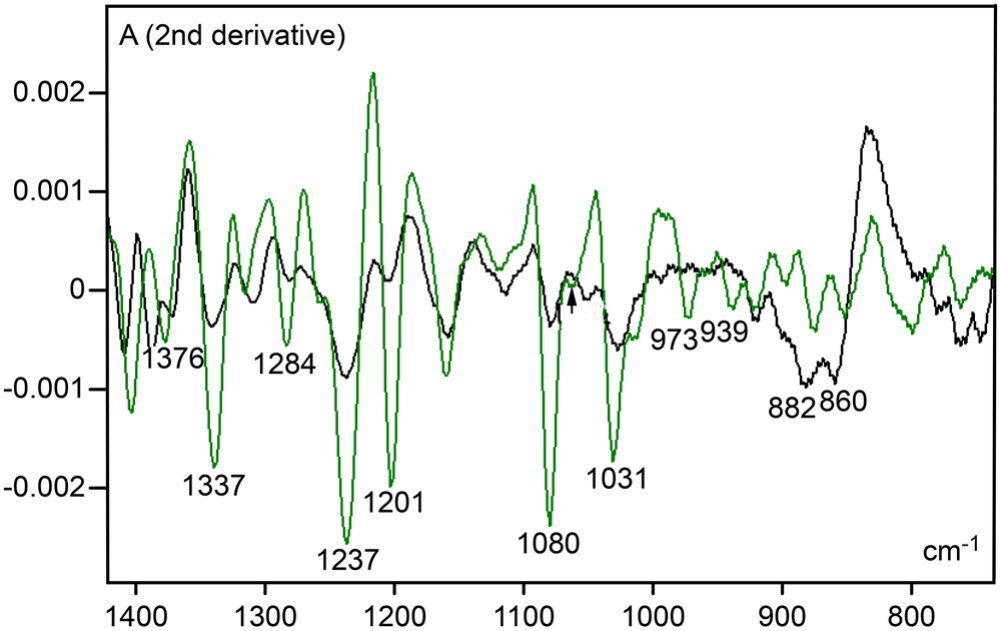
Savitzky-Golay’s 2nd-derivative spectra resolved with degree 2 and 11 smoothing points for cartilage ECM (black line) and collagen type II (green line)in the 1400–750 cm^-1^ spectral window. The arrow indicates 1062 cm^-1^ wavenumber position. A, absorbances.

The most reliable peaks for cartilage ECM in the l400-750 cm^-1^ spectral window, using the Savitzky–Golay’s 2nd-derivative, occurred at wavenumbers (882 and 860 cm^-1^) within the original 890–840 cm^-1^ spectral range (Figs 3 and 4) which is assigned to contribution of PrG carbohydrate vibration groups. No such peaks were highlighted in the collagen type II 2nd-derivative spectrum. A peak revealed at 1237 cm^-1^ in the original spectral profile of both the cartilage ECM and collagen type II (Fig 3) appeared highlighted after fitting the spectra to the 2nd-derivative (Fig 4). Intensification of this peak was conspicuous in the collagen type II spectrum (Fig 4). Another evident peaks at the 1400–750 cm^-1^ wavenumber range for collagen type II were verified at 1337, 1201 and 1080 cm^-1^ (Fig 4); the peak at 1337 cm^-1^ is assigned to vibration of -CH_2_ side chains [5, 25].

**Fig 5:**
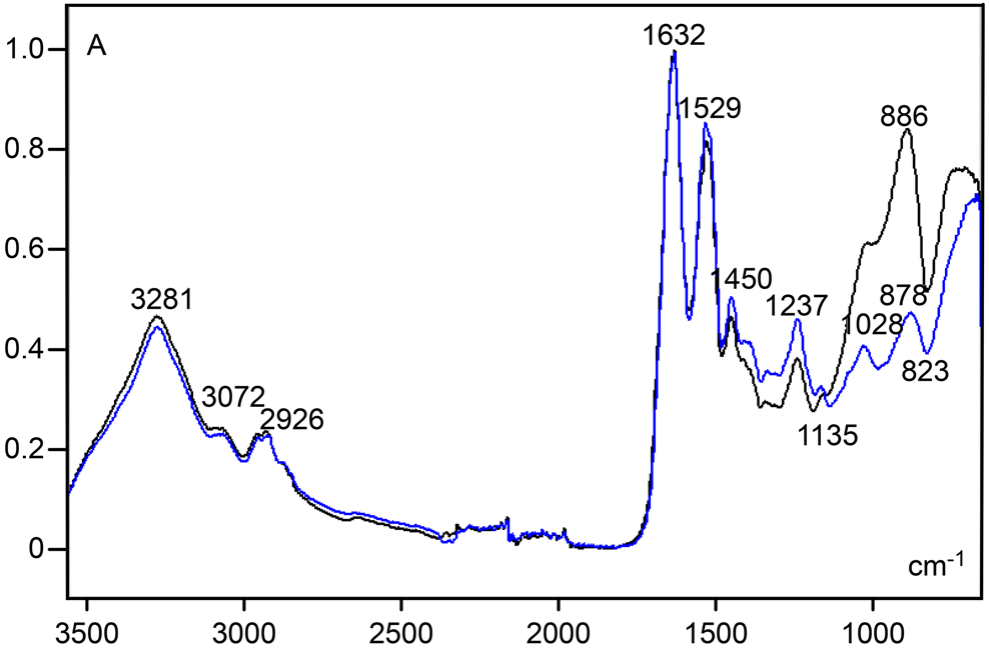
FT-IR spectral profiles for untreated (black line) and hyaluronidase-treated (blue line) ear cartilage ECM. The profiles were normalized with respect to the amide I peak. A, absorbances.

In contrast to findings for human and bovine articular cartilage [5, 6], a conspicuous peak at 1064–1062 cm^-1^, which is strongly contributed by sulfated GAGs, was not detected in the rat ear cartilage ECM (Fig 3), even when analyzing the 2nd-derivative spectrum obtained with degree 2 and 11 smoothing points (Fig 4). The same occurred when varying the calculation of the 2nd-derivative degree number from 2 to 4 and the number of smoothing points from 9 to 15. However, a deep reduction in absorbances at the 1135–700 cm^-1^ spectral window observed in the spectrum of the cartilage ECM subjected to hyaluronidase digestion (Fig 5, S2 Fig) is in agreement with the participation of carbohydrates in the rat ear cartilage ECM FT-IR spectrum. In the 1136/1135-824/823 cm^-1^ wavenumber range, two band peaks (1028–1027 and 884–878 cm^-1^) were revealed, and a decrease in band areas was evident both in spectra normalized to the amide I peak (Fig 5) or in hyaluronidase-treated ECM spectra which were not normalized with respect to this peak (S3 Fig. In this case, a slight height decrease of amide I and II band peaks was observed (S3 Fig), most likely on account of the contribution of PrG vibration groups to peaks most traditionally assigned to collagen content [5].

Peak fitting applied to the amide I region of cartilage ECM and collagen type II spectra revealed band peaks positioned at 1659, 1648, 1641, 1630 and 1620 cm^-1^; the largest area for the ECM was found for the band under the 1641 cm^-1^ peak, whereas that for collagen type II occurred under the 1630 cm^-1^ peak. After hyaluronidase digestion, the band peaks for the ECM shifted to 1650, 1641, 1630, 1620 and 1611 cm^-1^ and the largest areas were found under the 1641 and 1630 cm^-1^ peaks.

The effect of hyaluronidase digestion on GAG components of the ear cartilage ECM also reflected on AbRCam and ArRCam ratios (Table 1).

**Table 1:** Absorbance and area ratios obtained from FT- IR spectra of rat ear cartilage after hyaluronidase treatment. The spectra were normalized with respect to the amide I peak.

Identification	AbRCAm	ArRCAm
Cartilage ECM	0.810	1.40
Cartilage ECM after hyaluronidase digestion	0.474	0.35

AbRCAm, absorbance ratio corresponding to carbohydrate (886–878 cm^-1^)/amide I (1632 cm^-1^) band peaks; ArRCAm, area ratio corresponding to carbohydrate (1135–824 cm^-1^)/amide I (1728–1587 cm^-1^) spectral windows; ECM, extracellular matrix

**Fig 6:**
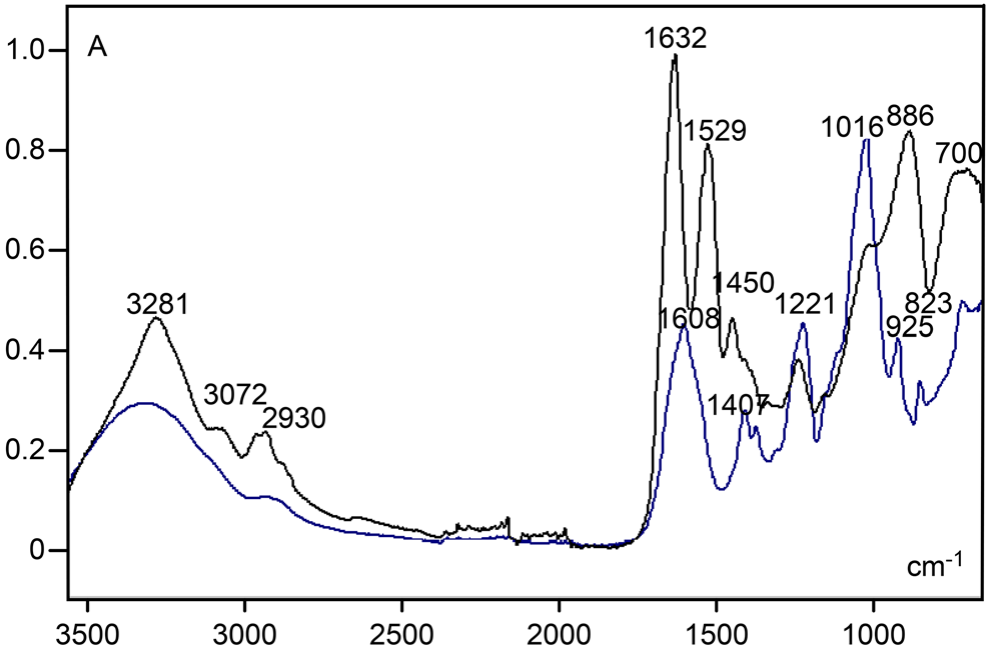
FT-IR spectral profile for the 4-CIS standard (navy line) compared to that for the ear cartilage ECM (black line). A, absorbances.

The FT-IR spectrum obtained for the 4-CIS standard, even when not fitted to normalization procedures, exhibited higher absorption intensities at the 1226–1221 cm^-1^ and 1025–1016 cm^-1^ band peaks compared to the spectrum of the cartilage ECM (Fig 6, S4A Fig). The highest peak occurred at the 1025–1016 cm^-1^ window, a spectral region which was characterized by decreased absorbances following hyaluronidase digestion (Fig 5), due to removal of sulfated GAGs. This peak was also very evident when 4-CIS and collagen type II spectra were compared to each other (S4 Fig B). The peak at 1226–1221 cm^-1^ in pure 4-CIS is assigned to vibration of GAG sulfate groups [26]. A peak at 1608–1606 cm^-1^, most likely attributed to–C = O stretching vibration [24], was also observed (Fig 6). Much smaller absorbances were evident at the 3400–2750 cm^-1^ spectral window, amide I and II regions and in the 1000–700 cm^-1^ wavenumber range of the 4-CIS standard compared to the cartilage ECM (Fig 6). The broad peak at ~3300 cm^-1^ and the low shoulder at ~2900 cm^-1^ in the 4-CIS spectral profile are potentially due to N-H stretching and O-H and C-H stretching vibrations [24], respectively. A low peak at 925–923 cm^-1^ was also revealed in the 4-CIS spectral profile (S4A Fig) relatively close to the frequency where an ECM spectral peak was detected (~890–886 cm^-1^).

**Fig 7:**
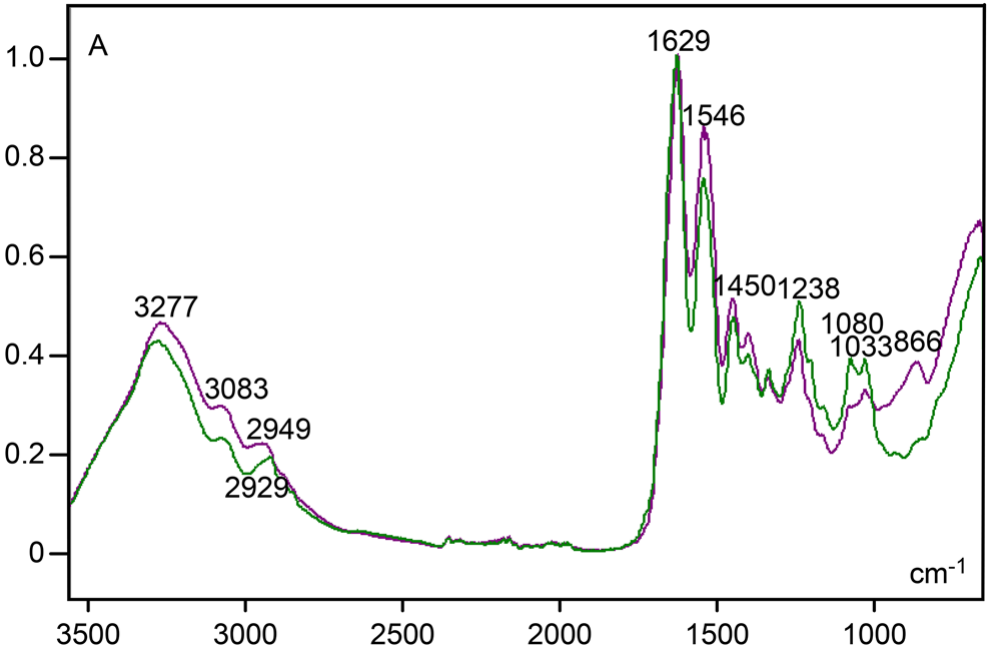
FT-IR spectral profile for dermal CB (purple line) compared to that for collagen type II (green line). A, absorbances.

When comparing the FT-IR spectrum of the ear dermal CB to that of collagen type II, similarities in absorption peak positions were detected at high frequencies and at regions corresponding to amides I, II and III (Fig 7). However, peaks at 1238–1237, 1080–1079 and 1033–1031 cm^-1^, which were evident in the collagen II spectrum (Figs 3 and 7), appeared with smaller absorbance values as peaks at 1238 and 1033 cm^-1^ and a shoulder at 1080 cm^-1^ in the spectrum of the dermal CB. However, absorbances at 866 cm^-1^ were much higher in the dermal CB than in collagen type II (Fig 7).

Summarized data on FT-IR band peak positions, absorbance heights and area units for cartilage ECM, collagen type II and dermal CB are shown in the S1 Table.

## Discussion

Our observations confirmed the relatively simple morphological structure of the rat ear cartilage as previously reported [8, 9]. However, to the best of our knowledge, present investigation was the first basic study to bring microspectroscopic FT-IR spectral characteristics, optical anisotropy and GAG histochemistry for rat ear cartilage into context. Although exhibiting a less complex structure than articular cartilage, ear cartilage ECM was found to contain macromolecularly oriented components, as demonstrated by its optical anisotropic properties.

The birefringence brilliance intensity of the ear CBs and cartilage ECM indicates variability on the ordered molecular supra-organization of collagen fibers and PrG and their aggregation and concentration. These properties were of a lower degree in the ECM compared to the dermal CBs. Highly ordered macromolecular organization as assessed by birefringence findings was also detected in the isolated collagen type II preparations used as a standard for the FT-IR study. This finding is consistent with previously reported data on the enthalpy and linear dichroism for this material [27] and for rat articular cartilage [28].

The intense metachromasy observed in the ear cartilage ECM was caused by the stacking of toluidine blue molecules bound electrostatically to stained GAG substrates [23, 28, 29]. Consequent to removal of hyaluronic acid and chondroitin-sulfates from ECM by testicular hyaluronidase, there were metachromasy abolishment and decrease in absorption intensity in the ~1140–820 cm^-1^ spectral range of the ECM FT-IR spectra. This spectral region is mostly assigned to vibration of carbohydrate, PrG and aggrecan groups, as previously reported for articular cartilage [1, 5, 6, 30].

In human and bovine articular cartilage, peaks that are assigned to PrG and glycoproteins (1376 cm^-1^) and to the –SO_3_^-^ symmetric stretching vibration of sulfated GAGs (1064 cm^-1^) in 2nd-derivative spectroscopy are sensitive to hyaluronidase digestion [5]. This digestion does not affect peaks at 1336 cm^-1^ and 1202 cm^-1^, which are mostly assigned to collagen [5, 7]. However, not even a low peak at 1064–1062 cm^-1^ was found after fitting the spectrum of the rat ear cartilage ECM to the Savitzky-Golay’s 2nd-derivative. Although only a modest peak was present in the collagen type II spectrum at 1376 cm^-1^, a peak at a slightly higher frequency was evident in the 2nd-derivative spectrum of the ear cartilage ECM. These results may be due to differences concerned with animal species [6] and/or cartilage types. The -SO_3_^-^ symmetric stretching vibration of sulfated GAGs at the 1064 cm^-1^ frequency in ear cartilage may be under-represented. On the other hand, contribution of -SO_3_^-^ asymmetric stretching vibration of these GAGs, usually reported to occur at ~1228 cm^-1^ [26], may have shifted in the ear cartilage ECM to ~1237 cm^-1^, where a peak was evident in the 2nd-derivative spectrum.

The decreased absorbance in the amide I band in non-normalized spectrum of the ear ECM after hyaluronidase digestion is consistent with reports demonstrating contribution of vibration of PrG chemical radicals to this band in articular cartilage [5]. In addition, changes in amide I band occur as PrG concentration increases in mixtures of collagen type II with increasing concentrations of aggrecan (Fig 1 A in [7]). These findings do not favor consideration of amide I for quantification of collagen II in cartilages.

When using the FT-IR peak fitting method, the largest area corresponding to the amide I band was under the 1641 cm^-1^ peak for cartilage ECM and under the 1630 cm^-1^ peak for collagen type II. This difference may reflect the effect of PrG protein moieties on collagen II FT-IR properties when these substances form a complex such as that in the ear cartilage ECM.

Several authors have used the amide I band region to obtain information on protein conformation, including that for collagen [31, 32]. Differences in the amide I/amide II ratio calculated for several proteins as albumin, β-lactoglobin and myoglobin have also been associated to variations in these proteins’ secondary or tertiary conformations [33]. Considering these reports and the finding that the amide I/amide II ratio for the ear cartilage ECM was lower than that for collagen type II, we may assume that the protein complex present in the ear cartilage ECM is arranged with a lower helicity level compared to pure collagen type II.

In the FT-IR spectral profile of the ear cartilage ECM, part of the amide III absorption appeared at the same frequency (1236–1225 cm^-1^) where 4-CIS shows a peak corresponding to the asymmetric stretching vibration of its SO_3_^-^ groups [5, 26]. Thus, it is reasonable to conclude that this peak in the spectrum for the ear ECM could not be as specific as the peak at 1027–1016 cm^-1^ (S4A and S4B Fig) for -SO_3_^-^ identification. We propose the latter for the evaluation of the –SO_3_^-^ contribution to the FT-IR spectral profile of the ear cartilage ECM.

Higher band heights in the FT-IR region assigned to carbohydrates (band peak at 890 cm^-1^) in spectra of collagen type I-containing dermal CBs, compared to the collagen type II standard, are probably due to the establishment of PrG-collagen complexes in CBs; decorin complexed with collagen I predominates in tendon CBs (Pimentel ER, personal communication). The intense birefringence brilliance in CBs highlights the highly ordered structure of these complexes, contributing to their FT-IR characteristics.

## Conclusions

1. Although structurally less complex than articular cartilage, ear cartilage contains oriented ECM macromolecular components.
2. Although collagen type II adds significantly to the FT-IR spectral profile of the rat ear ECM, the contribution of vibrations of carbohydrate radicals to this spectrum is also evident. GAG elements susceptible to hyaluronidase digestion play a role in the stereo-arrangement of the PrG-collagen II complex in the ear cartilage ECM and affects its FT-IR signature.
3. Considering the contribution of vibrations of PrG radicals to the collagen amide I IR spectral region, this region is not fully recommended for quantification of collagen type II in cartilages.
4. Based on amide I/amide II ratios and data from the literature, the protein complexes present in the ear cartilage ECM are assumed to be arranged with a lower degree of helical conformation than pure collagen II.
5. Compared to articular cartilage, the symmetric stretching of -SO_3_^-^ groups in ear cartilage, based on absorption at 1064 cm^-1^ appears under-represented. Because there is an overlap of the asymmetric stretching vibration of 4-CIS –SO_3_^-^ groups with the ECM amide III band (1236–1226 cm^-1^), a peak or even a shoulder at ~1025 cm^-1^ may be a better representative of the contribution of –SO_3_^-^ vibration groups to the FT-IR spectrum of the ear cartilage.
6. The higher IR absorption in the spectral region typical for vibration of carbohydrate groups (~870 cm^-1^) in the ear dermal CBs in comparison to collagen type II is most likely caused by PrG elements (e.g., decorin) binding to collagen type I.

## Supporting Information

S1 Fig Birefringence image of a pure lyophilized collagen type II mesh. After compensation of the birefringence brightness in the fine collagen fibers oriented from the SE to NW direction, enhancement of the birefringence brightness occurs in the fibers oriented in the opposite direction (SW to NE). Bar = 50 μm.
(TIF)

S2 Fig FT-IR spectral profiles for untreated (black line) and hyaluronidase-treated (blue line) ear cartilage ECM. Detail for the 1150–700 cm^-1^ spectral window. A, absorbances.
(TIF)

S3 Fig FT-IR spectral profiles for untreated (black line) and hyaluronidase-treated (pink line) ear cartilage ECM. The spectrum represented by the pink line, which was not normalized with respect to the amide I band, shows changes introduced by hyaluronidase treatment in amide I and II regions.
(TIF)

S4 Fig **FT-IR spectral profiles for ear cartilage ECM (A, black line), 4-CIS (A and B, navy line) and collagen II (B, green line).** Details for the 1450–700 cm^-1^ window. A (Y axis), absorbances.
(TIF)

S1 Table Numerical integral statistics applied to the FT-IR spectra obtained for rat ear cartilage and normalized to amide I using Grams software. (DOCX)
