# Supplementary figures and images for: FT-IR Microspectroscopy of Rat Ear Cartilage
Source: PLoS One. 2016 Mar 25;11(3):e0151989. doi: 10.1371/journal.pone.0151989 (PMC4807954; doi:10.1371/journal.pone.0151989)

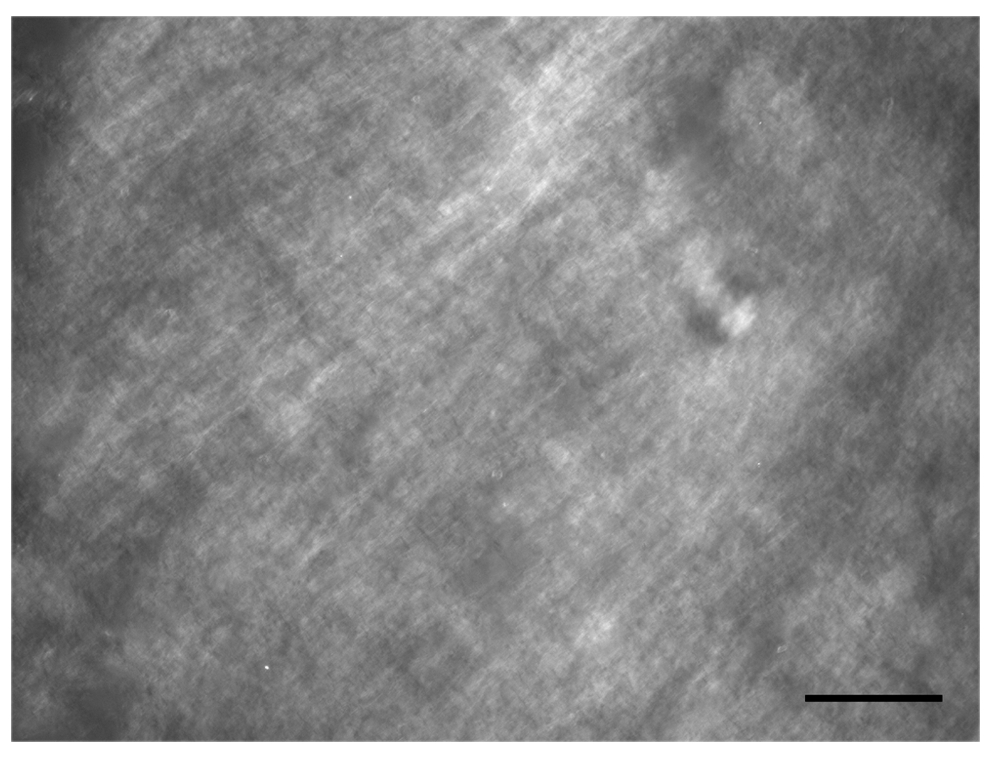

Supplement: S1 Fig — After compensation of the birefringence brightness in the fine collagen fibers oriented from the SE to NW direction, enhancement of the birefringence brightness occurs in the fibers oriented in the opposite direction (SW to NE). Bar = 50 μm. (TIF) [file pone.0151989.s001.tif]

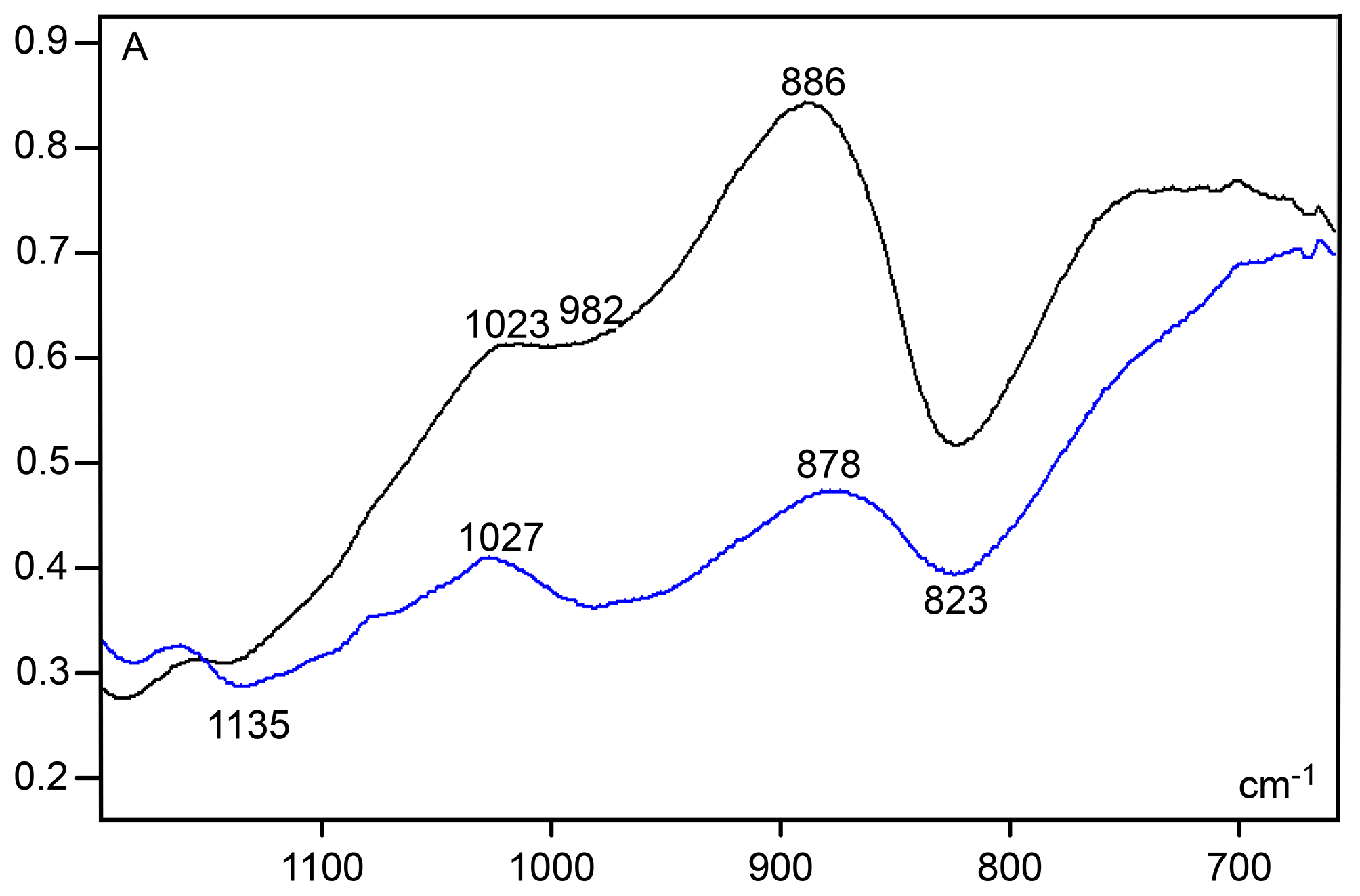

Supplement: S2 Fig — Detail for the 1150–700 cm-1 spectral window. A, absorbances. (TIF) [file pone.0151989.s002.tif]

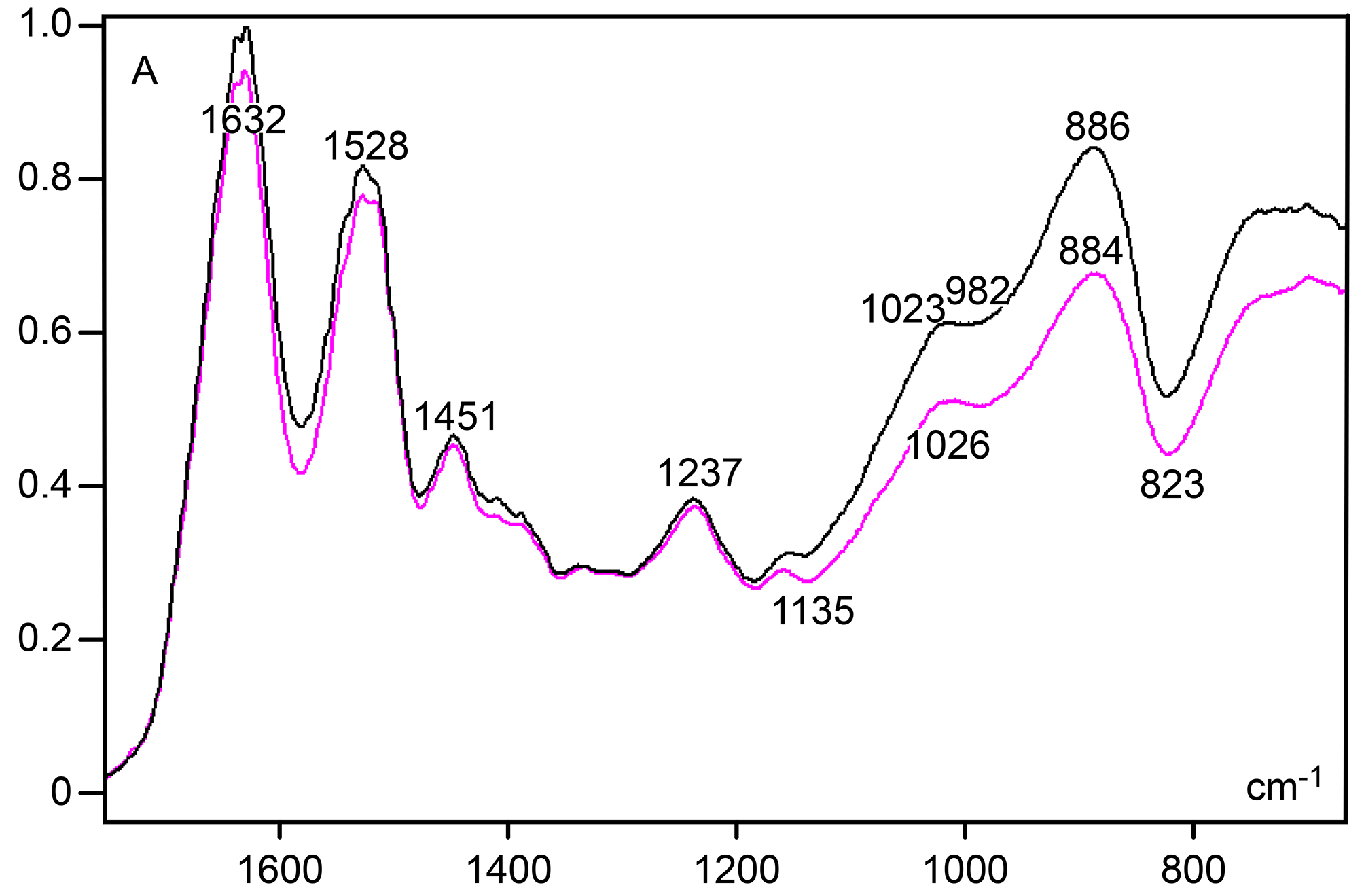

Supplement: S3 Fig — The spectrum represented by the pink line, which was not normalized with respect to the amide I band, shows changes introduced by hyaluronidase treatment in amide I and II regions. (TIF) [file pone.0151989.s003.tif]

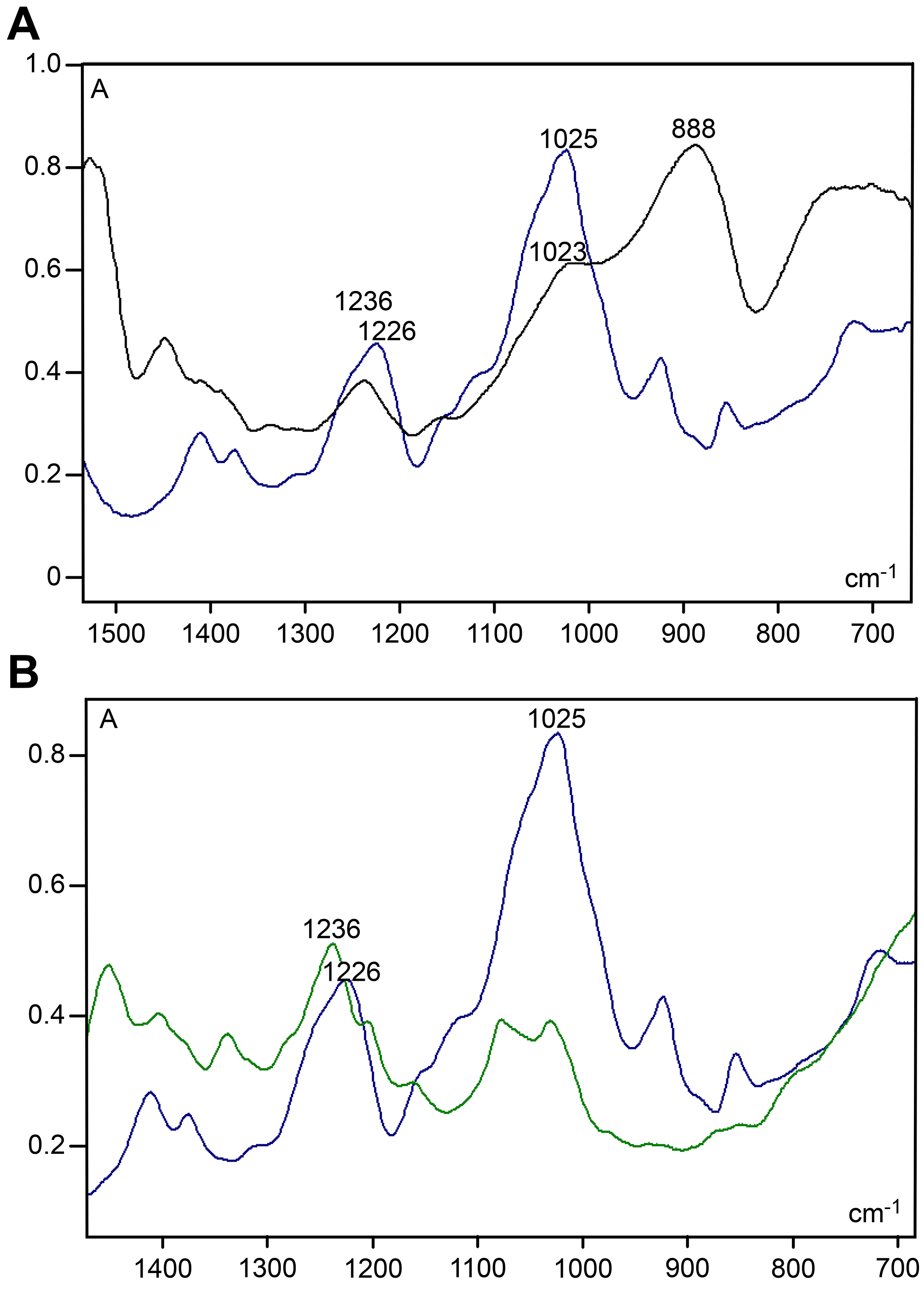

Supplement: S4 Fig — FT-IR spectral profiles for ear cartilage ECM (A, black line), 4-CIS (A and B, navy line) and collagen II (B, green line). Details for the 1450–700 cm-1 window. A (Y axis), absorbances. (TIF) [file pone.0151989.s004.tif]
