# Supplementary material for: FT-IR Microspectroscopy of Rat Ear Cartilage
Source: PLoS One. 2016 Mar 25;11(3):e0151989. doi: 10.1371/journal.pone.0151989 (PMC4807954; doi:10.1371/journal.pone.0151989)
Supplement: S1 Table — (DOCX) [file pone.0151989.s005.docx]

**S1 Table. Numerical Integral Statistics applied to the FT-IR spectra obtained for rat ear cartilage and normalized to amide I using Grams software.**

| **Identification** | **Band peak (cm^-1^)** | **Absorbance** | **Area units** | **Wavenumber edges (cm^-1^)** |
| --- | --- | --- | --- | --- |
| ECM amide I | 1632 (p) | 0.998 | 41 | 1737-1587 |
| ECM amide II | 1529-1528 (p) | 0.816 | 21 | 1579-1485 |
| ECM amide III | 1450 (p) | 0.466 | 2.4 | 1479-1417 |
| ECM c | 886 (p)  1020-1016 (s) | 0.838  0.611 | 61.8 | 1139-823 |
| ECM c after hyaluronidase | 878 (p)  1028-1027 (s) | 0.473  0.410 | 16. | 1132-823 |
| Collagen II amide I | 1630-1629 (p) | 1.000 | 41. | 1738-1587 |
| Collagen II amide II | 1547 (p) | 0.761 | 18. | 1584-1485 |
| Collagen II amide III | 1450 (p) | 0.476 | 4 | 1484-1426 |
| Collagen II c | 1079-1077 (p)  1032-1031 (p) | 0.400  0.400 | 13 | 1133-980 |
| CB amide I | 1628 (p) | 1.000 | 36 | 1721-1587 |
| CB amide II | 1546 (p) | 0.861 | 20 | 1585-1488 |
| CB amide III | 1451-1450 (p) | 0.515 | 4 | 1484-1426 |
| CB c | 866 (p) | 0.385 | 5. | 1000-840 |

c, carbohydrate groups; ECM, extracellular matrix; p, peak; s, shoulder; *, no normalization to amide I
